# Obstetrics

**Published:** 1859-05

**Authors:** 


### OBSTETRICS.

REPORT ON THE CASES OF OVARIOTOMY THAT HAVE OCCURRED IN GERMANY. By *Gustavus Simon.*—The statistics of ovariotomy hitherto published in Germany have been mixed up with English and American cases; and the object of this paper is to give an account of those operations which have been performed in Germany alone. As the operation has only become at all frequent there during the last ten years, it is still possible to obtain an exact account of the great majority of the patients, both as regards the operation itself and the ultimate fate of the individual. To make this as complete as possible, not only has the author referred to all the published cases, but has opened up a correspondence with the surgeons of all the chief towns of Germany. In this way he has collected sixty-one cases, twenty-three of which have not hitherto been published; and these must embrace the greatest part of those which have occurred, although it is still possible that some may have escaped notice. All foreign operations have been rigidly excluded, even when they have been performed by Germans in adjoining countries. The results of these researches are set forth in tables extending over from twenty to thirty pages. In these he gives not only the particulars of each operation as regards the mode of execution, the presence of adhesions, and the condition of the tumor, but also narrates the history of the patient both prior and subsequently to its performance, as also her later condition, whenever an account of this has been obtainable. Some of the cases, in spite of every care, remain defective in certain of these particulars.

The aim of the tables is to bring out as distinctly as possible the advantages or disadvantages of the operation; and the better to do this the cases of ovariotomy have been arranged under three divisions, viz., 1st, those in which the operation has been followed by a radical cure; 2d, those in which death has been the result; and 3d, those cases in which, although recovery has followed the operation, the advantage has proved transitory, doubtful, or non-existent. In this last category not only have been included those operations which, begun, have had to be abandoned on account either of the firmness of adhesions or the errors of diagnosis, but others in which, although the patient has recovered from a partially or completely executed ovariotomy, yet has died eventually from after-consequences of the same, or from the effects of the original disease. Thus, a case of Martin's has been so placed, in which the patient died eight months after a successful extirpation of a colloid cyst, owing to cancerous formations in the pancreas, lymphatic glands, and the lungs, which the operator himself regarded as connected with the original malady. So also with regard to a case of Küchenmeister's, wherein ovariotomy was left incomplete on account of the firmness of the adhesions, the patient recovering from the effects of the operation, but dying in three-quarters of a year from the rupture of a secondary cyst. Operations like these cannot be set down as radical cures, but must be regarded as of only transitory utility, if of any utility at all, for the prolongation of life. Although these patients felt themselves entirely well for some time after the operation, (in Martin's case for four months,) it is very questionable whether they would not have lived longer still had mere palliative punctures been resorted to. Under the same category must in future be placed those cases in which, after complete recovery from the operation, death still eventually takes place owing to the persistence or subsequent formation of abdominal fistulæ; those cases in which, very soon after the extirpation of medullary sarcoma of the ovary, cancerous formations appear in other organs; and finally, not only those cases in which secondary cysts spring up after incomplete extirpation, but also those where, after the extirpation of one diseased ovary, the cysts existing simultaneously in the other, forthwith increase and assume a similar threatening aspect. Each of these three primary divisions of the cases, derived from the issue of the operations, are formed into three subdivisions, dependent upon the nature of the operation and condition of the disease, viz., 1st, cases of ovariotomy completed; 2d, cases in which ovariotomy was attempted, but only incompletely performed or entirely abandoned on account of the strength of the adhesions; and 3d, cases in which, after the abdominal cavity had been opened, the diagnosis was found to be erroneous. This last subdivision, which does not strictly belong to the statistics of ovarian tumors, has nevertheless been retained, because, in judging of the danger of an operation, errors of diagnosis frequently cannot be avoided, and their consequences must not be lost sight of. It is not indeed likely, at the present period, that Dohlhoff's error, committed in 1838, of seeking to perform ovariotomy where no tumor of the abdomen existed, will be repeated; but it is continually happening that tumors of the uterus, retro-peritoneal tumors, etc., are confounded with ovarian tumors.

It is evident that the bearing of the statistical tables must be more exact upon German practice than those hitherto published in which English and American cases have played the chief part. In these we have only the results of the practice of German surgeons, (some of the most distinguished among these,) whose capability of forming the diagnosis and executing the operations can be appreciated. But it is believed that these new statistics are not only of more measurable value to the German practitioner than any that have preceded them, but may also serve even in other countries as a basis for forming a judgment concerning ovariotomy. If the tables already published comprise hundreds of cases, while these only related to sixty-one cases, yet the former were open to various important sources of error, which in these it has been sought, as far as possible, to avoid. Without referring to instances in which cases have been entered both under the names of the operator and the reporter, we may advert to two sources of error which must exert a good deal of influence on the conclusions to be drawn. The chief defect is that while cases which terminate favorably are made as public as possible, many cases having an unfortunate issue have been unrecorded. Of course it would be entirely unjustifiable for an operator to publish his successful and conceal his unsuccessful cases. But how many operators have only met with unsuccessful cases, and how many, after having published successful cases, have later met with only reverses! A great number of such cases would never be published at all; for while, on the one hand, we can scarcely blame an operator who has met with unsuccessful cases if he wait before publishing them until he has others of a more fortunate character to place by their side; so, on the other hand, ovariotomy has become so very common an operation, especially when unsuccessful, that an operator would incur the charge of tiresomeness if he published cases of failure of the operation, unless these exhibited special circumstances of great interest. How important these considerations are, however, in weighing the conclusions to be drawn from published cases, may be judged by examining R. Lee's statistical account, published in the thirty-fourth volume of the *Medico-Chirurgical Transactions.* With all his sources of information he was only able to indicate 51 operators, who had performed 162 operations; but in a country where practitioners are numbered by thousands, in which ovariotomy has been recognized as a permissible operation for twenty or thirty years, and where it has become so common that Clay has performed it 69 and Bird 31 times, can we believe that this operation, which neither requires dexterity acquired only by long practice, nor complicated instrumental apparatus, has only been performed by 51 persons? This is quite incredible; for in Germany, where the operation still has many and important opponents, there have been 34 operators for 61 operations. A second important source of error, vitiating the early statistics, consists in the enumeration among the cures, cases in which the operation had to be abandoned either on account of the adhesions or the faulty diagnosis. Now, although these women recovered, inasmuch as the disease still remained, and the patient had incurred the great danger due to gastrotomy, these cases should be regarded not as recoveries from, but failures of, the operation. It is true that Lee has sought to avoid this error, and abstracts 60 such cases from the 162 operations; but he found it impossible to give an account of the latest condition of the cases set down as cured. These two sources of error, Dr. Simon has taken every pains to avoid; and he believes his statistics claim much more confidence than Lee's, which neither comprised all the cases that had occurred, nor sufficiently controlled those they did comprise. These statistics also show that in Germany errors of diagnosis have not been of such frequent occurrence—operations not having been undertaken with the same rashness as in England and America. While in 60 of Lee's 162 cases the operation had to be abandoned, either on account of adhesions or errors in diagnosis, this was the case in only 17 of the 61 German cases.

The following is the summary of the results, as specified in the author's detailed tables: Of the 61 patients upon whom ovariotomy was either executed or attempted, 44 ($72\frac{8}{61}$ per cent.) died from the immediate effects of the operation; in 5 ($8\frac{2}{61}$ per cent.) it proved useless, or of only transitory benefit, although the patients recovered; and only in 12 patients ($19\frac{41}{61}$ per cent.) was a radical cure obtained. (The operation may even have been more unfavorable in Germany than here represented, as notwithstanding the search made for them other unsuccessful cases may not have been brought to light.) The operation was completely executed in 44 instances. Of these 44 there died 32 ($72\frac{8}{11}$ per cent.) from the immediate effects of the operation; one died eight months after in consequence of cancer in other organs; and 11 (25 per cent.) were radically cured. The operation was attempted in 15 cases, but on account of the firmness of the adhesions had to be abandoned or only partially performed. Of these, in one a radical cure followed, in three no advantage, or very transitory advantage, resulted, and in 11 death ensued as the immediate consequence of the operation. In two cases the operation was attempted; but the diagnosis was at fault; one of the patients died, and the other recovered.

These results are far less favorable than those which have been derived from statistics previously published; and while Fock and others place the danger of ovariotomy upon the same line as herniotomy and amputation of the thigh, it is, according to the above figures, more dangerous to life than the Cæsarean section, which is, according to Kaiser, attended with a mortality of 63 per cent., and, according to others, of about two-thirds of those upon whom it is performed.

In a postscript, Dr. Simon brings the cases up to 64 in number. In these a radical cure was effected in 12, in 6 the operation was at most of transitory utility, and in 46 it was followed by death.—*Scanzoni Beitrage zur Geburtskunde*, band iii. pp. 98, 142.—(*Medical Times and Gazette*, February 19, 1859.)

